# Stem Cells and Cardiac Repair

**DOI:** 10.1155/2015/153627

**Published:** 2015-05-07

**Authors:** Sadia Mohsin, Daniele Avitabile, Mohsin Khan

**Affiliations:** ^1^Cardiovascular Research Center, Temple University School of Medicine, Philadelphia, PA 19140, USA; ^2^Laboratory of Vascular Biology and Regenerative Medicine, Centro Cardiologico Monzino, IRCCS, 20138 Milan, Italy; ^3^Center for Translational Medicine, Temple School of Medicine, Temple University, Philadelphia, PA 19140, USA

Discovery of tissue specific stem cells capable of forming cardiac cell types has revolutionized cardiac medicine. Not long ago, cardiac tissue regeneration was considered an impossible task. The last ten years however has seen an explosion of cell based therapeutic approaches stimulating cardiac regeneration and in the process augmenting function in the heart following injury. Encouraging preclinical results have paved the way for clinical applications of cell therapy and preliminary results obtained from various clinical trials indicate that stem cell transplantation increases cardiac function comparable to the existing interventions for treatment of heart diseases. These are promising outcomes that indicate that the cells have the ability to modulate cardiac repair programs leading to replacement of the lost tissue. Nevertheless, the search continues for the optimal cell type that can promote “true cardiac regeneration,” supplement the lost cardiomyocytes, and at the same time form the angiogenic support structure. One of the first stem cell types to be used for cardiac regeneration was derived from the mononuclear fraction of the bone marrow and the cells were designated as bone marrow mononuclear cells [1]. Ensuing years saw establishment of the finding that the bone marrow is host to a variety of distinct stem/progenitor cell populations possessing cardiogenic repair potential. The study by D. Orlic et al. in the early part of the last decade showed that transplantation of lin^−^/c-kit+ cells effectively transdifferentiates into cardiac lineages leading to enhanced cardiac function after infarction [2]. At the same time, a large body of evidence pointed towards a role for bone marrow derived mesenchymal stem cells (MSCs) in the repair of the damaged heart [3]. Nevertheless, the search for a heart resident stem cell population continued till A. P. Beltrami et al. showed that the adult heart contains a resident stem cell population capable of differentiating into all three cardiac lineages (myocytes, endothelial, and smooth muscle cells) [4]. In addition to these widely studied stem cell types, researchers have used other extra cardiac stem cell types such as adipose derived stem cells [5], cortical bone derived stem cells [6], and cord blood stem cells for cardiac repair.

Despite the excitement, significant concerns persist around the ability of adoptively transferred cells to survive in the ischemic heart and some reports suggest as low as 1% of cells make it in the heart past the first few days of transplantation. Most of the salutary effects of cell therapy have been attributed to these few surviving cells and recent efforts have focused on boosting the survival, proliferation, and cardiac commitment of the donated stem cell population. This special issue contains a cluster of articles focused on augmenting ability of adoptively transferred stem cells to repair the damaged heart. X. Xue et al. demonstrated that genetic engineering of MSCs with Bcl-xl enhances survival and engraftment after transplantation leading to reduction in scar formation and increased functional recovery. Homing and migration of the adoptively transferred stem cells are another important determinant for the success of cell therapy. An interesting approach was employed using the ultrasound microbubble technique to promote MSC migratory ability to the infarcted myocardium. Authors show that ultrasound microbubble destruction increased MSC numbers in the infarcted heart mediated via SDF-1/CXCR4 axis. In contrast, the study by J. Liu et al. utilized adipose derived stem cells to show that curcumin, a naturally occurring food chemical, can promote angiogenic and survival ability of the cells augmenting their potential for the repair of ischemia reperfusion injury to the heart. Bone marrow derived endothelial progenitor cells (EPCs) represent a widely used cell type for cardiac regenerative therapies. EPCs have been shown to increase angiogenesis and augment cardiac function animal studies and undergoing clinical trials [7, 8]. EPCs remain one of the cells of choice for cardiac repair; however, the effect of different environmental factors remains to be fully elucidated. This special issue contains a very interesting finding about how low dose space-type ionizing radiation affects the long term cycling and survival of bone marrow derived EPCs opening up a host of possibilities in understanding how environmental factors influence stem cell function.

Additionally, controversy rages on whether the transplanted stem cells directly convert into cardiomyocytes or mediate their beneficial effects through the release of “paracrine effectors.” Characterization of the stem cell secretome has provided some interesting clues about how factors released by the cells modulate cellular processes in target cells. These paracrine factors include growth factors, chemokine, cytokines, various proteins, and small molecules. Recently, small microvesicles released by stem cells under different physiological conditions have been included in the definition for paracrine factors. Exosomes are tiny vesicles released by the cells, carry proteins, mRNAs, and microRNA, and have the ability to modulate cellular and molecular signaling processes [9]. A number of recent studies have highlighted that exosomes derived from stem cells possess cardiac repair potential and are able to recapitulate the salutary effects of cell therapy promoting cardiac morphological and physiological functions [10, 11]. In this special issue, MSC derived exosomes and their role in cardiac regeneration have been highlighted by a report and a detailed review on MSC secretome. The study by K. Kang et al. demonstrates the therapeutic value of exosomes derived from CXCR4 overexpressing MSCs for the repair of heart after myocardial infarction. In addition, the review by C. Gallina et al. on MSC secretome and its potential application for cardiac regeneration is a wonderful collection of past and current research on MSC secreted factors and extracellular vesicles

We hope the readers will find this special issue interesting and a timely effort covering current issues and advancements in the field of stem cell based therapies for the repair of damaged heart.

*Sadia Mohsin* *Sadia Mohsin* *Daniele Avitabile* *Daniele Avitabile* *Mohsin Khan* *Mohsin Khan*
